# The Role of Partial Agonists and Specifically Cariprazine in Dual Disorders

**DOI:** 10.1192/j.eurpsy.2024.461

**Published:** 2024-08-27

**Authors:** Á. Barabássy, Z. B. Dombi, R. Csehi, D. Djuric, G. Németh

**Affiliations:** Gedeon Richter Plc., Budapest, Hungary

## Abstract

**Introduction:**

The treatment of dual disorders, the co-occurrence of a major psychiatric disorder and a substance use disorder, represents a great challenge. Recent articles recommend antipsychotics with a dopamine partial agonism as first line treatment for these patients. Studies also postulate that drugs targeting the dopamine D3 receptors specifically might have an advantage, as these receptors are involved in drug-related reward, drug-seeking, and drug-intake behaviour. One compound that has both, partial agonist- and D3- activity is cariprazine.

**Objectives:**

To evaluate the real-world evidence of the effectiveness of cariprazine in patients with dual disorders.

**Methods:**

We performed a systematic literature search on PubMed, looking for English language articles published between January 2017 - September 2023 with the following search terms: (cariprazine) AND (psychosis OR schizophrenia OR schizoaffective OR bipolar depression OR bipolar mania OR bipolar disorder OR major depressive disorder) AND (“substance use disorder” OR cocaine OR alcohol OR cannabis OR heroin OR “double diagnosis” OR “dual diagnosis”) NOT (animal OR rat OR mouse) NOT (review or meta-analysis). An additional targeted hand search of congress reports, posters, and case reports was also conducted.

**Results:**

The search yielded 8 articles with 11 case reports. Mental health disorders included psychosis, schizophrenia, schizoaffective disorder, PTSD, and bipolar disorder while the abused substances were methamphetamine, cannabis, alcohol, and cocaine. All case reports described an improvement in both the symptoms of mental and substance use disorder with reduced craving and drug use and in some cases even ceasing drug use all together.

**Conclusions:**

In summary, evidence suggests that cariprazine seem to be a potential candidate for dual disorders as it improves symptoms of both mental and substance use disorders.

**Disclosure of Interest:**

Á. Barabássy Employee of: Gedeon Richter Plc., Z. Dombi Employee of: Gedeon Richter Plc., R. Csehi Employee of: Gedeon Richter Plc., D. Djuric Employee of: Gedeon Richter Plc., G. Németh Employee of: Gedeon Richter Plc.

